# Cannabidiol Exerts Anticonvulsant Effects Alone and in Combination with Δ^9^-THC through the 5-HT1A Receptor in the Neocortex of Mice

**DOI:** 10.3390/cells13060466

**Published:** 2024-03-07

**Authors:** Yasaman Javadzadeh, Alexandra Santos, Mark S. Aquilino, Shanthini Mylvaganam, Karolina Urban, Peter L. Carlen

**Affiliations:** 1 Krembil Research Institute, University Health Network, Toronto, ON M5S 0T8, Canadapeter.carlen@uhnresearch.ca (P.L.C.); 2 Institute of Biomedical Engineering, University of Toronto, Toronto, ON M5S 1A1, Canada; 3 Avicanna Inc., Toronto, ON M5G 1V2, Canada; 4 Department of Medicine (Neurology), University Health Network, Toronto, ON M5G 2C4, Canada

**Keywords:** drug resistant epilepsy, cannabinoids, serotonin, 5-HT1A receptor, electrophysiology, cannabidiol (CBD), delta-9 tetrahydrocannabinol (Δ^9^-THC), anticonvulsant

## Abstract

Cannabinoids have shown potential in drug-resistant epilepsy treatment; however, we lack knowledge on which cannabinoid(s) to use, dosing, and their pharmacological targets. This study investigated (i) the anticonvulsant effect of Cannabidiol (CBD) alone and (ii) in combination with Delta-9 Tetrahydrocannabinol (Δ^9^-THC), as well as (iii) the serotonin (5-HT)1A receptor’s role in CBD’s mechanism of action. Seizure activity, induced by 4-aminopyridine, was measured by extracellular field recordings in cortex layer 2/3 of mouse brain slices. The anticonvulsant effect of 10, 30, and 100 µM CBD alone and combined with Δ^9^-THC was evaluated. To examine CBD’s mechanism of action, slices were pre-treated with a 5-HT1A receptor antagonist before CBD’s effect was evaluated. An amount of ≥30 µM CBD alone exerted significant anticonvulsant effects while 10 µM CBD did not. However, 10 µM CBD combined with low-dose Δ^9^-THC (20:3 ratio) displayed significantly greater anticonvulsant effects than either phytocannabinoid alone. Furthermore, blocking 5-HT1A receptors before CBD application significantly abolished CBD’s effects. Thus, our results demonstrate the efficacy of low-dose CBD and Δ^9^-THC combined and that CBD exerts its effects, at least in part, through 5-HT1A receptors. These results could address drug-resistance while providing insight into CBD’s mechanism of action, laying the groundwork for further testing of cannabinoids as anticonvulsants.

## 1. Introduction

Epilepsy is a disorder characterized by spontaneous recurrent seizures and over 30% of patients display drug-resistance [1]. Not only do these patients suffer from a lower quality of life, but they are also at higher risk of complications including sudden unexpected death in epilepsy (SUDEP) [2]. Thus, drug-resistance remains a prominent obstacle in epilepsy treatment, emphasizing the critical need for the discovery of novel therapeutics. 

The therapeutic potential of phytocannabinoids, such as Cannabidiol (CBD) or delta-9 tetrahydrocannabinol (Δ^9^-THC), has been a research area of interest for many centuries. Unlike Δ^9^-THC, CBD lacks psychoactive effects and has been shown to possess great therapeutic potential in the management of many diseases [3], particularly childhood epilepsy.

In vitro studies have shown that CBD reduces neuronal excitability as well as burst amplitude and duration [4,5,6,7]. In vivo studies using various epileptic models have also shown CBD’s anticonvulsant potential by attenuating seizure severity and mortality [4,7,8,9]. Furthermore, clinical studies, examining the efficacy of CBD as an anticonvulsant, have displayed a reduction in seizure frequency and severity with the use of CBD [10,11,12,13]. However, many of these trials use different compounds with varying purities, making comparisons of results between trials difficult, and patients in these trials are using CBD in combination with other anti-epileptic drugs, confounding the results. Additionally, some clinical trials observed initial improvement in seizures with CBD but seizures worsened after a short time [14]. Currently, Epidiolex is a medication that was approved by the United States Food and Drug Administration (FDA) for patients with Dravet syndrome and Lennox–Gastaut syndrome. Epidiolex is a pure CBD product, and although it has been shown to be effective, further studies are required to expand our knowledge regarding its use for non-pediatric epilepsy and other epilepsy types [15,16]. Nevertheless, CBD does display promising anticonvulsant effects, high tolerability, and low toxicity, further supporting its safe use as a therapeutic [17,18].

In vitro and in vivo studies of the potential anticonvulsant effect of Δ^9^-THC have been controversial, as some studies show an anticonvulsant effect whereas others showed either no effect or even a proconvulsant effect [19,20,21]. Studies conducted in hippocampal slices have shown that 0.1 µM Δ^9^-THC enhanced excitability whereas 1 µM Δ^9^-THC depressed excitability [22]. In addition to the inconclusive effects of Δ^9^-THC, the compound also displays psychoactive properties and adverse side effects, making its independent use in the clinical setting unattractive as a therapeutic [23].

Recently, many parents have chosen to try alternative therapies for their children struggling with drug-resistant epilepsy. Anecdotal accounts of CBD-enriched cannabis have shown great therapeutic benefits in children with epilepsy. The mother of a 5-year-old girl with Dravet syndrome, who was experiencing up to 50 bilateral tonic-clonic seizures per month, chose to start her child on a strain of cannabis that had a high CBD and low Δ^9^-THC concentration. This CBD-enriched cannabis reduced her daughter’s seizure frequency >90% [24]. Since then, many surveys of parents who have used CBD-enriched cannabis as a therapy for their child’s epilepsy have been conducted [25,26]. For example, one survey showed that 85% of all parents reported a reduction in seizure frequency, with 14% reporting seizure freedom after the use of CBD-enriched cannabis [27]. As such, much interest has been shown towards the therapeutic benefits of the combination of different phytocannabinoids. Various animal models of epilepsy have shown that the addition of small amounts of Δ^9^-THC improves the effectiveness of CBD [28,29]. Clinical trials using combinations of CBD and Δ^9^-THC at various ratios observed significant improvements in seizure frequency and quality of life; thus, concluding that CBD-enriched cannabis extracts are potentially anticonvulsant as an add-on treatment in children with drug-resistant epilepsy [30,31]. Conversely, other studies have shown the combination of CBD and Δ^9^-THC to be proconvulsant. In a trial on the effect of Sativex (1:1 CBD: Δ^9^-THC) on patients with Multiple Sclerosis, some patients reported their ‘first ever seizures’ [32]. Overall, the evidence for the beneficial effects of CBD in combination with Δ^9^-THC remain insufficient and further studies are required before implementation in the clinical setting [33]. 

The etiology of epilepsy is complex and multi-factorial [34]. Thus, the ideal anticonvulsant drug would be one that engages multiple targets to help re-balance electrical activity within the brain. The literature has implicated a multitude of different targets as the mechanism of action of CBD [35]. Here, we focus on the serotonergic system, but it is reasonable to assume that CBD exerts its anticonvulsant effects through a combination of pharmacological targets [6,36,37,38,39,40].

The role that the serotonergic system plays in epilepsy has been highly studied. The serotonin (5-HT)1A receptor has attracted much attention as it is linked to a K^+^ channel, allowing the hyperpolarization of neurons. A study using a Mg^2+^-free model of epileptiform activity found that the addition of a selective 5-HT1A receptor agonist decreased the population spike amplitude in the CA1 hippocampal region [41,42]. In vivo studies have also shown similar results. Epileptic animal models displayed a reduction in seizure frequency after the 5-HT1A receptor was stimulated with an agonist [41,43,44]. Due to the anticonvulsant effects of 5-HT1A receptor stimulation, it has been speculated that CBD could exert its anticonvulsant effects through this receptor. Using binding analysis experiments, it was shown that CBD is an agonist at the 5-HT1A receptor [45]. 

Many studies have shown that CBD exerts anticonvulsant effects through the 5-HT1A receptor [46,47,48]; however, other contradicting studies showed that the anticonvulsant effects observed by CBD were not through the 5-HT1A receptor [49]. Thus, further studies are required to clarify the role that the 5-HT1A receptor plays in CBD’s mechanism of action. 

The aims of this study were to (i) investigate the anticonvulsant potential of CBD alone and (ii) in combination with Δ^9^-THC on mouse cortical slices made epileptic, and to (iii) determine whether CBD exerts its anticonvulsant effects through the 5-HT1A receptor.

## 2. Materials and Methods

### 2.1. Animal Preparation

Experiments were conducted on juvenile C57BL/6 mice (Charles River Laboratories, Wilmington, MA, USA) of either sex, post-natal day 14–21. All animal experiments and procedures were approved by the University Health Network Animal Care Committee (protocol AUP 750.50) and carried out in accordance with guidelines outlined by the Canadian Council of Animal Care (CCAC). A total of 63 mice were used for all experimentation and an average of 2 cortical slices were used from each animal. Mice were chosen as their brains are small in size and thus, it is more likely that connectivity between neurons remain intact during slice preparation [50]. Additionally, juvenile mice were chosen as they have shown to be more susceptible to developing seizures in comparison to adults [51,52], and this age correlates with an age range in children wherein cannabinoids have been most studied to date. It has been shown that CBD has beneficial effects in pediatric epilepsies and focal cortical dysplasia is the most common cause of drug-resistant pediatric epilepsy [14,53,54]. Thus, cortical slices from juvenile mice were used to best model pediatric epilepsy. 

### 2.2. Cortical Slice Preparation

Mice were first anesthetized with an intraperitoneal injection of 50 mg/kg sodium pentobarbital. The pedal reflex was used to ensure that mice were deeply anaesthetized before quickly decapitating the mice. The whole brain was rapidly removed and transferred to a sucrose dissection solution (in mM: 248 sucrose, 26 NaHCO_2_, 10 D-glucose, 2 KCl, 3 MgSO_4_, 1.25 H_2_NaPO_4_, 1 CaCl_2_) at 4 °C. The cerebellum and forebrain were removed, and a cyanoacrylate adhesive gel was used to fix the brain to a block. Using a Leica 1200 V vibratome, 500 μm thick cortical slices were prepared and hemisected. Slices were incubated in artificial cerebrospinal fluid (ACSF, in mM: 123 NaCl, 25 NaHCO_2_, 10 D-glucose, 3.5 KCl, 1.3 MgSO_4_, 1.2 HNaPO_4_, 1.5 CaCl_2_; pH adjusted to 7.4 with 95% O_2_, 5% CO_2_) for 30 min at 37 °C, followed by 1 h at room temperature in the perfusion chamber prior to recordings. 

### 2.3. Electrophysiology

During recordings, each slice was placed in a submerged recording chamber and was perfused at 10 mL/min with ACSF at 35 °C and aerated with 95% O_2_, 5% CO_2._ A borosilicate capillary glass electrode (1.5 mm, World Precision Instruments, Sarasota, FL, USA) was filled with ACSF and used to record local field potentials (LFP). This LFP electrode (~2 MΩ resistance) was positioned in cortical layers 2/3. Using a Multiclamp 700B amplifier (Molecular devices), a Digidata 1322A digitizer (Axon Instruments, Burlingame, CA, USA), and the PClamp software (version 10.2) (Axon Instruments/Molecular Devices Corporation, San Jose, CA, USA), signal acquisition was conducted. 

### 2.4. Materials

Seizures were induced using 4-aminopyridine (4-AP), a blocker of K^+^ channels, which causes prolonged glutamate release with excitation and consequent epileptiform activity. 4-AP is a well-founded model of epilepsy in mice, as is demonstrated by the induction of prolonged recurrent seizures without ‘exhaustion’ of seizure activity overtime in recordings. Thus, the ability of CBD to suppress 4-AP-induced seizures is useful for examining CBD’s efficacy as an anticonvulsant. The 4-AP was dissolved in double-distilled water to create a 100 mM stock solution, was aliquoted in 1 mL tubes and stored at −20 °C. On experimental days, the 4-AP was thawed and diluted in ACSF to a final concentration of 100 µM.

For aim 1, CBD was provided by Avicanna in a powdered form. Working solutions of CBD were freshly prepared daily prior to experiments. CBD was dissolved in dimethyl sulfoxide (DMSO; Sigma, St. Louis, MO, USA) to create the stock solution before being diluted in ACSF to create the final concentrations of 10, 30, or 100 µM. The concentrations that were seen to be effective were higher than therapeutic concentrations previously shown in the literature [5,55]; however, 4-AP is a very robust model of status epilepticus [56,57] and is more difficult to treat, justifying the anti-seizure benefits potentially observed at higher concentrations.

For aim 2, a CBD isolate and delta-9-tetrahydrocannabidiol (Δ^9^-THC) distillate were donated from Avicanna (Toronto, Canada) in a self-emulsifying drug delivery system comprised of TWEEN80. To prepare the stock solutions, the CBD and Δ^9^-THC solutions were sonicated and final concentrations of 10 mM CBD and 0.5, 1.5, or 2.5 mM Δ^9^-THC were made, aliquoted into 1 mL amber tubes, and stored at −4 °C. On experimental days, the stock solutions were diluted in ACSF to create a final solution of 10 µM CBD:0.5 µM Δ^9^-THC (20:1 ratio), 10 µM CBD:1.5 µM Δ^9^-THC (20:3 ratio), or 10 µM CBD:2.5 µM Δ^9^-THC (20:5 ratio).

For aim 3, The 5-HT1A receptor antagonist and agonist used were *N*-[2-[4-(2-Methoxyphenyl)-1-piperazinyl]ethyl]-*N*-2-pyridinylcyclohexanecarboxamide maleate (WAY100635) and (±)-8-Hydroxy-2-dipropylaminotetralin hydrobromide (8-OH-DPAT), respectively (Tocris Bioscience, Ellisville, MO, USA). Stock solutions of 10 µM WAY100635 and 10 mM 8-OH-DPAT were created by dissolving the compounds in sterile water before storing at −20 °C. On experimental days, these solutions were thawed and diluted in ACSF to a final concentration of 10 nM WAY100635 and 10 µM 8-OH-DPAT. 

### 2.5. Protocol

**Figure 1:**
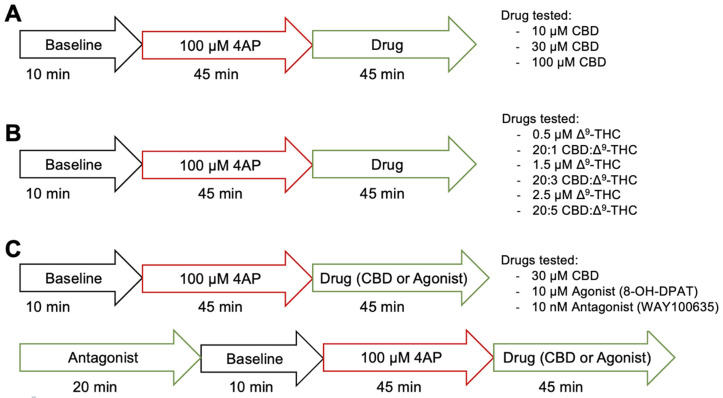
Illustrative protocol of electrophysiological experimentation. (**A**) Protocol of aim #1, assessing the anticonvulsant effects of 10, 30, or 100 µM CBD. (**B**) Protocol of aim #2, assessing the anticonvulsant effect of 0.5, 1.5, or 2.5 µM Δ^9^-THC alone or the combination of 20:1, 20:3, or 20:5 CBD:Δ^9^-THC. (**C**) Protocol of aim #3, assessing the role of 5-HT1A receptors in CBD’s mechanism of action using 10 µM 8-OH-DPAT, a 5-HT1A receptor agonist and 10 nM WAY100635, a 5HT1A receptor antagonist.

Figure 1 illustrates the protocol of the electrophysiological experimentation. For aims 1 and 2, we assessed the anticonvulsant effects of CBD alone and in combination with low dose Δ^9^-THC, after seizure induction with 4-AP, respectively. An initial 10-min baseline recording was taken. Afterwards, 100 μM 4-AP dissolved in ACSF was added for ~45 min to reliably induce seizure activity, before a 15-min recording was taken. Then, depending on the treatment condition, various concentrations of CBD, Δ^9^-THC, or combinations of CBD and Δ^9^-THC dissolved in ACSF with 4-AP were added for ~45 min, after which another 15-min recording was taken. For aim 1, the drug in the treatment conditions was either 10 (n = 7), 30 (n = 10), or 100 (n = 11) µM CBD (Figure 1A). For aim 2, the drug(s) in the treatment condition was either a combination of 20:1 (n = 5), 20:3 (n = 10), or 20:5 (n = 9) CBD:Δ^9^-THC, or 0.5 (n = 5), 1.5 (n = 7), or 2.5 (n = 10) µM Δ^9^-THC alone (Figure 1B).

In aim 3, we assessed the role that the 5-HT1A receptor plays in CBD’s mechanism of action (Figure 1C). A positive control experiment was first conducted in which an initial 10-min baseline recording was taken. An amount of 100 μM 4-AP dissolved in ACSF was added for ~45 min to reliably induce seizure activity, before a 15-min recording was taken. Then, 10 µM 8-OH-DPAT, a 5-HT1A receptor agonist, dissolved in ACSF with 4-AP was added for ~45 min, after which another 15-min recording was taken (n = 8). In another condition, to confirm that the anticonvulsant effects of 8-OH-DPAT were exerted through the 5-HT1A receptor, slices were pre-treated with 10 nM WAY100635, a 5-HT1A receptor antagonist, for 20 min before adding 100 μM 4-AP dissolved in ACSF with WAY100635 for ~45 min and taking a 15-min recording. Then, 10 µM 8-OH-DPAT dissolved in ACSF with WAY100635 and 4-AP was added for ~45 min, after which another 15-min recording was taken (n = 8) to see if pre-treatment with the 5-HT1A receptor antagonist would abolish the effects of the 5-HT1A receptor agonist. For the treatment condition, an initial 10-min baseline recording was taken before pre-treating the cells with 10 nM WAY100635. Then, 100 μM 4-AP dissolved in ACSF with the WAY100635 was added for ~45 min to reliably induce seizure activity, before a 15-min recording was taken. Afterwards, 30 µM CBD dissolved in ACSF with WAY100635 and 4-AP was added for ~45 min, and a 15-min recording was taken (n = 6).

For all studies, a vehicle control condition was conducted to ensure that DMSO or TWEEN80 as the vehicle did not impact the results of this study. Analysis of acute experiments with these vehicles showed that the addition of DMSO or TWEEN80 after seizure induction did not impact any of the features of interest; thus, neither vehicle impacted the results observed in this study. 

### 2.6. Data and Statistical Analysis

Data analyses were initially performed using pClamp 10.2 and MATLAB software, version R2020b. LFP recordings were filtered using a low-pass filter at 1250 Hz and reduced by a factor of 10. A 60 Hz notch filter with three harmonics was applied to eliminate noise before applying a high-pass filter at 0.25 Hz. The features of interest measured included duration (in seconds), amplitude (in mV), coastline length/second, and frequency of seizure-like events as well as duration (in seconds) and frequency of inter-ictal bursting events. Seizure-like events were defined as excitable activity lasting longer than five seconds, typical for 4-AP-induced seizures. Amplitude was defined as the difference between the highest and lowest points within the seizure-like event. Coastline length is the sum of the distance (absolute change in voltage of the signal) for a seizure-like event. Coastline length was divided by duration of that seizure-like event to provide a measure of burst intensity/second. Coastline length/second is indicative of seizure intensity with higher values representing more intense seizures. Frequency was defined as the average number of events in the last 10 min of each recording. Inter-ictal bursting events were defined as any excitable activity that was less than five seconds in duration and occurred after the end of one seizure-like event but before the start of another. Features of interest were measured for the last 10 min of each recording and averaged. Statistical analysis was performed using Graphpad (Version 9). Each sample size (n) equated to a single brain slice and sample sizes subjected to statistical analysis had at least five samples per group. For each experiment, a Wilcoxon’s matched paired two-tailed *t*-test was used to compare the mean features of interest of the drug condition in each slice to the pre-drug control condition in the same slice. For aim 3, a Mann–Whitney unpaired two-tailed *t*-test was also used for analyses across conditions to compare pre-treated with antagonist conditions to not pre-treated conditions. Results were considered significant when *p* < 0.05. For graphing purposes, percent change from pre-drug conditions were calculated using this formula:

% change = ((mean drug condition − mean pre-drug control condition)/mean pre-drug control condition) × 100.

## 3. Results

### 3.1. AIM #1—Anticonvulsant Effect of CBD Alone

#### Extracellular Effect of CBD on Seizure-Like Events (SLE) and Inter-Ictal Bursting Events

**Figure 2:**
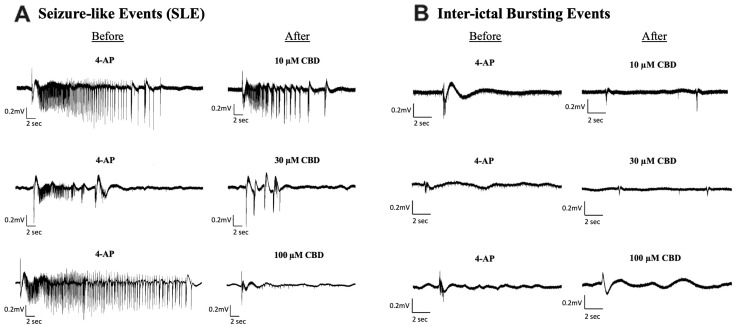
Sample traces of seizure-like events and inter-ictal bursting events before and after the addition of Cannabidiol (CBD). Example of electrophysiological recordings of (**A**) seizure-like events (SLE) and (**B**) inter-ictal bursting events before (**left column**) and after (**right column**) the addition of 10 µM, 30 µM, and 100 µM CBD in 3 separate slice experiments.

**Figure 3:**
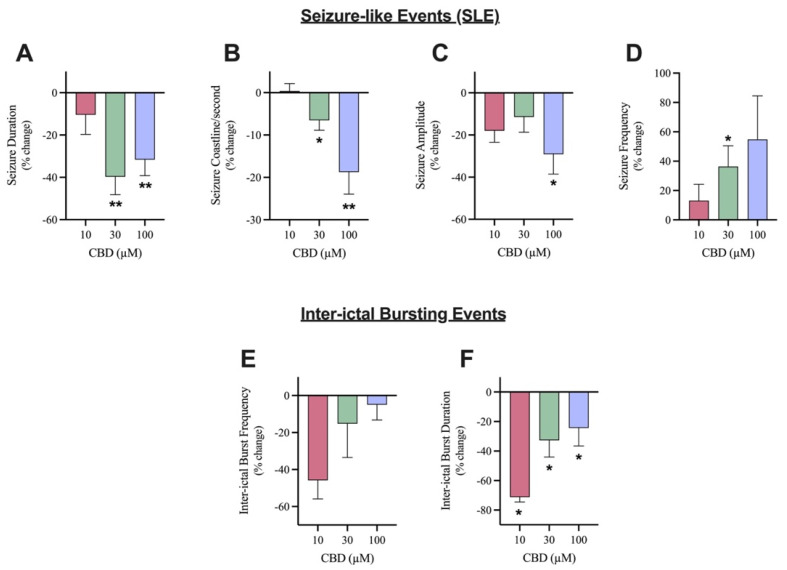
Comparison of the effect of Cannabidiol (CBD) on seizure-like events and inter-ictal bursting events. A bar graph comparing the effect of 10 µM (n = 7), 30 µM (n = 10), and 100 µM (n = 11) CBD on the mean (**A**) duration (in seconds), (**B**) coastline/second, (**C**) amplitude (in mV), and (**D**) frequency of seizure-like events and (**E**) frequency and (**F**) duration (in seconds) of inter-ictal bursting events in layer 2/3 of mouse cortical brain slices after seizure induction with 4-aminopyridine (4-AP). Data is displayed as average % change from pre-CBD (4-AP only) condition ± SEM. A Wilcoxon’s matched paired two-tailed *t*-test was used for all analyses to compare the CBD condition in each slice to the pre-CBD (4-AP only) condition in the same slice. Each sample size (n) equated to a single brain slice. * *p* < 0.05, ** *p* < 0.01. No statistical difference is left blank.

The effects of 10, 30, and 100 µM CBD on SLE (Figure 2A) as well as inter-ictal bursting events (Figure 2B) were examined. The addition of 10 µM CBD did not significantly alter any of the features of interest after seizure induction (Figure 3A–D). Although not significant, 10 µM CBD addition did display reductions in SLE duration (10.4% reduction) and amplitude (17.9% reduction) (Figure 3A,C). Significant anticonvulsant effects were observed at ≥30 µM CBD. The addition of 30 µM CBD caused a significant effect on the duration, coastline/second, and frequency of SLEs (Figure 3A,B,D) but no significant difference in the amplitude (Figure 3C). After the addition of 100 µM CBD, a significant decrease was observed in duration, coastline/second, and amplitude of SLEs (Figure 3A–C). Comparing the higher concentrations of CBD tested, 30 µM CBD application reduced SLE duration by 39.7% while 100 µM CBD application reduced SLE duration by 31.6% (Figure 3A). Alternatively, 100 µM CBD showed a larger reduction in seizure intensity, indicated by the coastline/second measurement (Figure 3C). Only 30 µM CBD displayed a significant increase in seizure frequency (Figure 3D), showing more frequent SLEs of lower duration, intensity, and amplitude. 

There were no significant changes in the frequency of inter-ictal bursting events at any of the concentrations tested (Figure 3F). Although not significant, it is noteworthy that 10 µM CBD application reduced frequency of inter-ictal bursting events by 45.8% while 30 µM and 100 µM CBD reduced frequency of inter-ictal bursting events by 15.2% and 5%, respectively. However, there was a significant decrease in the duration of inter-ictal bursting events at all concentrations tested (Figure 3E). Surprisingly, the addition of 10 µM CBD displayed the largest reduction as the duration of inter-ictal bursting decreased by 71.2%, whereas the 30 µM CBD and 100 µM CBD conditions demonstrated a 32.67% and 24.39% reduction, respectively. 

### 3.2. AIM #2—Anticonvulsant Effect of CBD Combined with Δ^9^-THC

#### Extracellular Effect of CBD Combined with Δ^9^-THC on SLE

**Figure 4:**
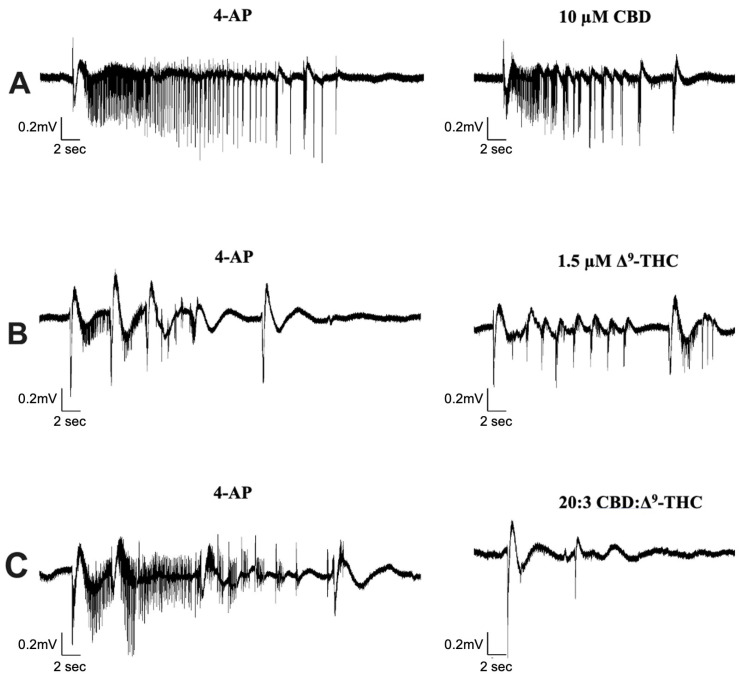
Sample traces of seizure-like events in cannabinoid combination studies. Example of electrophysiological recordings of seizure-like events before (**left column**) and after (**right column**) the addition of (**A**) 10 µM CBD, (**B**) 1.5 µM Δ^9^-THC, and (**C**) 20:3 CBD: Δ^9^-THC in 3 separate slice experiments.

**Figure 5:**
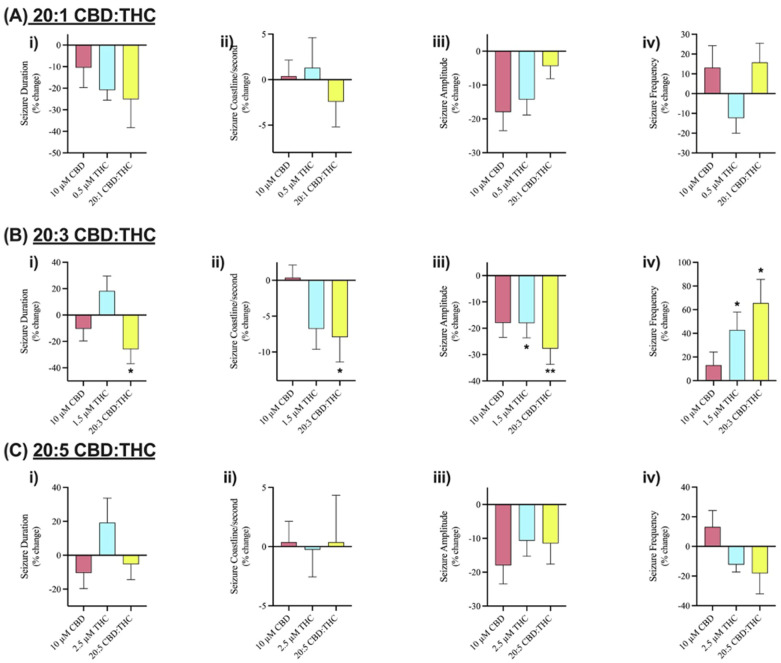
Comparison of the effect of Cannabidiol (CBD) and delta-9 tetrahydrocannabinol (Δ^9^-THC) alone and in various combinations on seizure-like events. Bar graphs in row (**A**) compare the effect of 10 µM CBD (n = 7), 0.5 µM Δ^9^-THC (n = 5), and 20:1 CBD:Δ^9^-THC (n = 5). Row (**B**) compares the effect of 10 µM CBD (n = 7), 1.5 µM Δ^9^-THC (n = 7), and 20:3 CBD:Δ^9^-THC (n = 10). Row (**C**) compares the effect of 10 µM CBD (n = 7), 2.5 µM Δ^9^-THC (n = 10), and 20:5 CBD:Δ^9^-THC (n = 9). Within each combination of CBD and Δ^9^-THC, (**i**) duration (in seconds), (**ii**) coastline/second, (**iii**) amplitude (in mV), and (**iv**) frequency of seizure-like events in mouse cortical brain slices after seizure induction with 4-aminopyridine (4-AP) was analyzed. Data is displayed as average % change from pre-drug (4-AP only) condition ± SEM. A Wilcoxon’s matched paired two-tailed *t*-test was used for all analyses to compare the drug condition in each slice to the pre-drug (4-AP only) condition in the same slice. Each sample size (n) equated to a single brain slice. * *p* < 0.05, ** *p* < 0.01. No statistical difference is left blank.

We investigated the effect of CBD combined with Δ^9^-THC in a 20:1, 20:3, and 20:5 ratio. Figure 4 displays sample traces of CBD and Δ^9^-THC combined in a 20:3 ratio. Application of 0.5 µM or 2.5 µM Δ^9^-THC had no significant effects on seizure activity (Figure 5A,C), whereas 1.5 µM significantly decreased seizure amplitude and increased seizure frequency (Figure 5B, panel iii–iv).

The literature using a mouse model of Dravet Syndrome found that the effects of low-dose Δ^9^-THC are enhanced when combined with a sub-anticonvulsant dose of CBD [58]. Results from aim #1 displayed nonsignificant reductions in duration and amplitude of seizure-like events and inter-ictal burst frequency after the addition of 10 µM CBD. Thus, 10 µM CBD was combined with 0.5, 1.5, or 2.5 µM Δ^9^-THC to create 20:1, 20:3, or 20:5 CBD: Δ^9^-THC ratios. The effect of these various ratios on seizure-like events were investigated. 

The results demonstrated no significant anticonvulsant effects on SLEs after the addition of 10 µM CBD in any of the features of interest (Figure 3A–D). Similarly, low doses of Δ^9^-THC (0.5, 1.5, or 2.5 µM) did not show any significant effects on the features of interest either (Figure 5). Combining these phytocannabinoids in a 20:1 ratio displayed greater reductions in seizure duration and coastline/second than either compound alone (Figure 5A, panels i–ii). Comparably, a 20:5 ratio displayed greater reductions in seizure frequency than either phytocannabinoid alone (Figure 5C, panel iv). However, the results observed after the addition of 20:1 or 20:5 ratio were not statistically significant. 

Significant reductions in duration, coastline/second, and amplitude of seizure-like events were observed after the addition of CBD and Δ^9^-THC in a 20:3 ratio (Figure 4 and Figure 5B, panels i–iii). Once again, these effects were greater than CBD or Δ^9^-THC alone. For example, the addition of 10 µM CBD displayed a 10.4% and 17.9% decrease in seizure duration and amplitude, respectively. However, the 20:3 ratio of CBD: Δ^9^-THC significantly reduced burst duration and amplitude by 25.9% and 27.7%, respectively (Figure 5B, panels i and iii). Similarly, a 20:3 ratio of CBD to Δ^9^-THC showed a significant increase in frequency of seizure-like events by 65.6% compared to a 13.1% increase after CBD alone (Figure 5B, panel iv).

Thus, 10 µM CBD combined with low doses of Δ^9^-THC has greater anticonvulsant effects than CBD alone with significant effects demonstrated at a 20:3 ratio of CBD: Δ^9^-THC. 

### 3.3. AIM #3—CBD’s Mechanism of Action

#### Extracellular Effect of Blocking 5-HT1A Receptor on SLE

**Figure 6:**
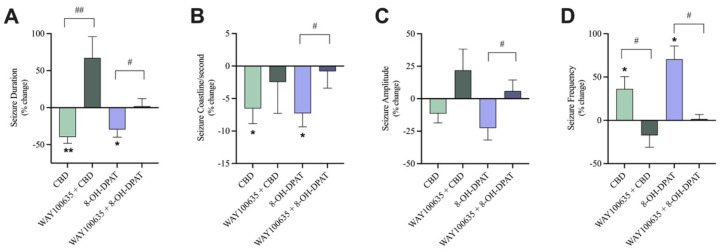
The effect of pre-treatment with a 5-HT1A receptor antagonist on seizure-like events. A bar graph comparing the effect of 30 µM CBD (n = 10) with 30 µM CBD pre-treated with 10 nM WAY100635, a 5-HT1A receptor antagonist (n = 6) on the (**A**) duration (in seconds), (**B**) coastline/second, (**C**) amplitude (in mV), and (**D**) frequency of seizure-like events in mouse cortical brain slices after seizure induction with 4-aminopyridine (4-AP). As the positive control condition, 10 µM 8-OH-DPAT, a 5-HT1A receptor agonist (n = 8), was compared to 10 µM 8-OH-DPAT pre-treated with 10 nM WAY100635 (n = 8). Data is displayed as % change from pre-drug (4-AP only or 4AP + WAY100635) conditions ± SEM. A Wilcoxon’s matched paired two-tailed *t*-test was used for all analyses to compare the drug condition in each slice to the pre-drug condition in the same slice. * *p* < 0.05, ** *p* < 0.01. A Mann–Whitney unpaired two-tailed *t*-test was used for analyses across conditions to compare pre-treated with antagonist conditions to not pre-treated conditions. # *p* < 0.05, ## *p* < 0.01. Each sample size (n) equated to a single brain slice. No statistical difference is left blank.

As a positive control, 8-OH-DPAT, a 5-HT1A receptor agonist displayed similar anticonvulsant results to 30 µM CBD, with significant reductions in seizure duration and coastline/second and significant increases in seizure frequency (Figure 6A,B,D). Pre-treatment with WAY100635, the 5-HT1A receptor antagonist, abolished 8-OH-DPAT’s effects as there was a significant increase in the percent change of seizure duration, coastline/second, and amplitude and a significant decrease in the percent change of seizure frequency in the pre-treated with antagonist condition compared to the not pre-treated conditions (Figure 6A–D). 

As previously discussed, addition of 30 µM CBD significantly reduced the duration and coastline/second and significantly increased the frequency (Figure 6A,B,D) of seizure-like events. 

Blocking the 5-HT1A receptor with WAY100635 before the addition of 30 µM CBD significantly increased the percent change of seizure duration (Figure 6A) in the pre-treated with antagonist condition compared to the not pre-treated condition while the percent change of seizure frequency significantly decreased (Figure 6D). Blocking the 5-HT1A receptors did generally reduce CBD’s effect on coastline/second and amplitude of seizure-like events; however, these trends were not significant. When the 5-HT1A receptors were not blocked, CBD displayed a 6.5% and 11% decrease in coastline/second and amplitude, respectively. Conversely, when 5-HT1A receptors were blocked, CBD’s effects were attenuated, displaying a 2.4% decrease and 21.9% increase in coastline/second and amplitude, respectively (Figure 6B,C).

Thus, pre-treatment with WAY100635 dampened the effect of 30 µM CBD on coastline/second and amplitude of seizure-like events and significantly abolished the effects of 30 µM CBD on the duration and frequency of seizure-like events. 

## 4. Discussion

### 4.1. The Anticonvulsant Effect of CBD Alone

The results of this study showed that CBD alone did display anticonvulsant effects, especially at concentrations greater than 30 µM. Interestingly, although the reductions in SLE duration after 30 µM and 100 µM CBD application were both significant, the results displayed a larger reduction in SLE duration after 30 µM CBD (39.7% reduction) compared to 100 µM CBD (31.6% reduction) addition. However, only 100 µM CBD significantly reduced burst amplitude. These findings are in line with the previous literature stating CBD’s potential biphasic effect depending on the feature of interest [7,59]. 

Overall, after the addition of CBD, we observed more frequent seizures of less intensity. Patients with epilepsy are often prescribed a combination of medications; thus, to reduce adverse side effects, lower doses are preferred. Although 10 µM CBD did not display any significant effects on SLEs, a general anticonvulsant trend was observed through decreases in duration and amplitude of SLEs. It was particularly interesting that 10 µM CBD only caused significant reductions in the duration of inter-ictal bursting events. A dose of 10 µM CBD could exert seemingly nonsignificant anticonvulsant effects through both ictal and inter-ictal characteristics. This compares to the literature showing that certain compounds are more largely involved in the generation of inter-ictal events than ictal events [60]. Further studies are required to fully elucidate the potential anticonvulsant effect of CBD.

4-AP is a well-founded model of epilepsy in mice, as is demonstrated by the induction of prolonged recurrent seizures without ‘exhaustion’ of seizure activity over time in recordings. Thus, the ability of CBD to suppress 4-AP-induced seizures, after the fact, is useful for examining CBD’s efficacy as an anticonvulsant. However, patients more often use medications to prevent, rather than treat, seizures. It is important to note this limitation of our study. Although we investigated the potential of CBD as a treatment of SLEs, our study did not investigate the ability of CBD to prevent SLEs induced by 4-AP. The efficacy of CBD pre-treatment as an anticonvulsant would strengthen the argument for translation into clinical settings, where CBD could potentially prevent prolonged seizures. Further experimentation on the anticonvulsant effect of CBD pre-treatment is needed to elucidate this neuroprotective capacity.

### 4.2. The Anticonvulsant Effect of CBD Combined with Δ^9^-THC

Currently, Epidiolex is a medication approved by the United States Food and Drug Administration (FDA) for patients with Dravet syndrome and Lennox–Gastaut syndrome. Epidiolex is a pure CBD product and although it has been shown to be effective, anecdotal reports have shown that adding other phytocannabinoids is more efficacious in seizure control than CBD alone [24,27]. Considering that the first experiments revealed the anticonvulsant potential of 10 µM CBD through nonsignificant reductions SLEs and inter-ictal bursts, we then examined the potential anticonvulsant interactions between CBD and Δ^9^-THC after seizure induction with 4-AP. 

Two important conclusions were drawn from these results. Firstly, the addition of low dose Δ^9^-THC to a low concentration of CBD potentiates the effect of CBD to a therapeutic level. Secondly, when comparing the various ratios tested, only a 20:3 ratio of CBD:Δ^9^-THC demonstrated significant anticonvulsant effect on SLEs. These results suggest that in all features of interest, 10 µM CBD and 1.5 µM Δ^9^-THC display greater anticonvulsant effects in combination than either compound does alone. 

To our knowledge, there are a limited number of studies that have looked at the interactions of CBD and Δ^9^-THC in in vitro brain slices made epileptic. One in vitro study using a muscarinic agonist-induced epilepsy model within the piriform cortex of rats showed that CBD and Δ^9^-THC combined did not have greater anticonvulsant effects than Δ^9^-THC alone [61]. These disparities could be due to differences in brain area, the model of epilepsy, or cannabinoid concentrations. Our work parallels in vivo studies which found that the effects of low-dose Δ^9^-THC are enhanced when combined with a subtherapeutic CBD dose [28,29,58]. However, it was also found that chronic use of this combination had proconvulsant effects and increased premature mortality rates [58]. Our study used acute slices and thus, we were unable to examine the long-term effects of a 20:3 CBD: Δ^9^-THC combination. Another study showed a synergistic effect between CBD and Δ^9^-THC only in a 1:1 ratio while the 5:1 ratio displayed a partial non-synergistic effect [62]. Thus, lower doses of Δ^9^-THC relative to CBD did not produce the same potentiating effects that our results displayed. This could be due to differences in species, possibly indicating a species-specific effect when combining CBD and Δ^9^-THC. 

Although the literature shows no consensus regarding the therapeutic use of CBD and Δ^9^-THC combined, many drug-resistant patients often turn to CBD-enriched cannabis products. Surveys and studies on these patients show a great reduction in seizure frequency and severity [25,26,63]. However, due to Δ^9^-THC’s psychoactive effects, the compound is clinically unattractive as an independent therapeutic. The safety profile is an important consideration when investigating novel drugs. Unlike Δ9-THC, which has been associated with states of psychosis [64,65], many studies have shown that CBD is well tolerated across many doses. In both short- and long-term administration, no concerning adverse effects were seen on the central nervous system or vital signs when using CBD, including high doses [18]. A study carried out on patients with drug-resistant epilepsy displayed CBD’s long-term safety and tolerability [66]. Thus, CBD also displays high tolerability and low toxicity through its independent use. We showed that lower doses of Δ^9^-THC can potentiate low CBD doses in vitro. Not only are lower doses associated with less adverse side effects, but CBD has also been shown to ameliorate the psychoactive effects of Δ^9^-THC through various mechanisms [67,68,69,70,71,72]. Through these mechanisms, CBD and Δ^9^-THC combined at low doses have great therapeutic potential as we can harness their individual anticonvulsant effects without the potential adverse effects, providing a solution for the reluctance to use Δ^9^-THC in the clinical setting. Our results are important as they parallel what is observed clinically, further justifying more research into the interactions of CBD and Δ^9^-THC. 

### 4.3. The Role of 5HT1A Receptors in CBD’s Mechanism of Action

There has been much research shedding light on the role that the serotonergic system plays in seizures [73,74]. Fenfluramine is a recently approved drug that has high efficacy for the treatment of seizures in Dravet syndrome and Lennox–Gastaut syndrome [75]. Studies have shown that fenfluramine exerts its anticonvulsant effects by enhancing serotonergic transmission through various receptors, including the 5-HT1A receptor [44,76]. Here, we investigated whether CBD exerts its anticonvulsant effects through the 5-HT1A receptor.

After the addition of 8-OH-DPAT, a selective 5-HT1A receptor agonist, we observed significant reductions in the features of interest, leading to the conclusion that 5-HT1A receptor stimulation can exert anticonvulsant effects. The literature supports our results as many studies have observed similar anticonvulsant effects after 5-HT1A receptor stimulation [41,42,43,77]. The anticonvulsant effect of 8-OH-DPAT was very similar to the effect observed after 30 µM CBD. Binding analyses experiments have shown that CBD is a known 5-HT1A receptor agonist, and a multitude of evidence has shown that the 5-HT1A receptor is a pharmacological target for CBD’s other therapeutic effects [45,78,79,80]. Thus, it is imaginable that the 5-HT1A receptor could be a target for CBD’s anticonvulsant effects. Our results provide evidence for this as pre-treatment with a 5-HT1A receptor antagonist abolished the anticonvulsant effects of CBD. In vivo studies have similarly shown that CBD’s anticonvulsant effects are abolished by a 5-HT1A receptor antagonist [46,47]. Conversely, a pentylenetetrazol seizure rat model displayed that CBD’s anticonvulsant effects are independent of the 5-HT1A receptor, but these differences could be due to the dosing and animal model that was used [49]. Knowledge on CBD’s mechanism of action is important to determine the patient population in which this new potential therapeutic is most effective as well as optimal dosing by monitoring the effect on the target pathway in the patient. 

Additionally, 5-HT1A receptor stimulation as a potential mechanism by which CBD exerts its anticonvulsant effects is a very promising result, especially for individuals with drug-resistant epilepsy, as these patients are at a higher risk for sudden unexpected death in epilepsy (SUDEP) [2]. Studies using SUDEP animal models have shown decreased firing from serotonergic neurons during and after seizures, implicating the serotonergic system in SUDEP [81,82]. Other studies using a SUDEP animal model showed that stimulation of the serotonergic system inhibited seizure-induced respiratory arrest [83]. Thus, not only does CBD have potential to be a novel anticonvulsant drug that improves seizure control but could also reduce the risk of SUDEP. However, many more studies are required to fully comprehend the role that the serotonergic system plays in SUDEP and how to harness its therapeutic potential.

## 5. Conclusions

This study shows that CBD, especially at higher doses, displays anticonvulsant effects on mouse brain neocortical slices after seizure induction with 4-AP. The anticonvulsant effects of lower doses of CBD can be potentiated with the addition of low-dose Δ^9^-THC, suggesting that the combination of these phytocannabinoids, specifically at a 20:3 CBD: Δ^9^-THC ratio, can have greater anticonvulsant effects than either phytocannabinoid alone. Additionally, CBD’s anticonvulsant effects were abolished when slices were pre-treated with a 5-HT1A receptor antagonist. Thus, CBD exerts its anticonvulsant effects, at least in part, through the 5-HT1A receptor.

In conclusion, these results help address the barrier of drug-resistance while providing insight into CBD’s mechanism of action, laying the groundwork for further testing of cannabinoids as anticonvulsants.

## 6. Patents

The outcomes of this study have resulted in a successful patent of the cannabinoid formulation with Avicanna Inc. for reducing the incidence of seizures and sudden unexpected death in epilepsy.

## Data Availability

The data that support the findings of this study are available from the corresponding author upon reasonable request.
